# Commentary: Factors Influencing Implementation of Family-Centered Care in Neonatal Intensive Care Units

**DOI:** 10.3389/fped.2021.741958

**Published:** 2021-09-22

**Authors:** Andréia Cascaes Cruz, Mavilde L. G. Pedreira, Myriam Aparecida Mandetta

**Affiliations:** ^1^Departamento de Enfermagem Pediátrica, Escola Paulista de Enfermagem, Universidade Federal de São Paulo, São Paulo, Brazil; ^2^National Council for Scientific and Technological Development CNPq, Ministry of Science, Technology and Innovation, Brasilia, Brazil

**Keywords:** family-centered care, parents, children, infants, neonatology, neonatal intensive care

## Introduction

Although strides have been made in the global implementation of family-centered care (FCC), progress has been uneven and many advancements are still incipient—sometimes rendering FCC implementation an elusive dream. The principal finding of the study conducted by Oude Maatman et al. (1) was the recognition by healthcare teams of parents as primary caregivers, a critical element in FCC practice and parental involvement in the care of their infants. The investigators recommended starting FCC implementation by changing the mindset of healthcare teams toward families in neonatal intensive-care units (NICUs).

In Brazil, FCC implementation in NICUs faces a number of barriers: (i) health literacy is typically poor among families, who also tend not to be fully aware of their rights; (ii) healthcare teams often have long-held constraining beliefs (family not viewed as primary caregiver in NICU; family presence deemed to overstimulate infants or increase infection risks; parents seen as visitors, not partners); (iii) many healthcare teams are unfamiliar with the concept and practice of FCC; (iv) poor infrastructure precludes accommodating families in NICUs; (v) heavy workloads deprive nursing teams of having time to involve families in infant care (2–5). Although these and other barriers may stem from different backgrounds across the globe (6), in Brazil they result primarily from socioeconomic inequality, poor education, and the overall culture within the healthcare system.

## The Sources of the Problem

Being together with the neonate in a NICU and sharing decision-making with the healthcare team are core principles of FCC, and a family right. Poverty, health illiteracy, and other social inequities put families at a disadvantage when faced with healthcare situations. They should be supported by the NICU healthcare team to be involved with care provision, which requires being listened to with dignity and respect. Promotion of inclusion and equity constitute a crucial nursing approach in order to reduce current disparities in Brazilian healthcare. Emphasis should be placed on investing in health literacy to empower families through education, improving their “capacity to obtain, process, and understand basic health information and services needed to make appropriate health decisions” (7).

Regardless of socioeconomic status, however, having a baby admitted to a NICU is often a daunting experience (8), causing family members to feel pressured, vulnerable, deprived of autonomy, and unable to express their needs. This overwhelming experience often leads family members to avoid complaining or arguing, due to the fear of being denied entrance to the NICU.

## Families as Partners

Research has shown that Brazilian nurses diverge in their understanding of FCC practice, and grasping its core aspects has proven insufficient to ensure its implementation (3). The prevailing view is that FCC concerns the ability of family members to provide day-to-day technical care and assist staff in its provision, while other professionals believe FCC is related to providing emotional support (2). Although participation is an essential concept in FCC, many Brazilian professionals are not familiar with this approach, and most Brazilian hospitals lack FCC policies. Overcoming the prevailing lack of knowledge regarding FCC requires investment in education, sensitizing of healthcare teams, and preparing them to engage in a partnership with families (9).

In a paternalistic ethical approach, decisions about treatment are often restricted to members of the healthcare team. Family autonomy is not respected, and shared decision-making is not even considered (10). A productive strategy might involve the inclusion of both theoretical and hands-on FCC content in the curricula of undergraduate, graduate, and permanent education programs.

European healthcare professionals have underscored that FCC success is influenced by the facilities available at the NICU ward, which should include essential services that parents might need on a daily basis, such as a canteen or kitchen, laundry room, and places where they can temporarily rest and step away from the NICU environment (1). Family inclusion can be influenced by both the physical setting and welcoming available at the NICU.

A study carried out in 10 neonatal units in Brazilian public hospitals revealed that seven institutions provided private space for families, either within or outside neonatal units, but in four hospitals these spaces were reserved for mothers only (5). Overall, however, the most frequent type of accommodation in NICUs consisted of a single chair, as opposed to several beds. Only one hospital had a bedside recliner to accommodate the mother or father for long stays (5). Single rooms, private restrooms, or even a place to sleep remain a “dream” for Brazilian parents (2, 4, 5).

Although the implementation of FCC-guided practices is hindered by infrastructure deficiencies, this is not the primary factor. These hurdles should rather be a reason for healthcare professionals and managers to implement FCC in NICUs, and thus positively transform the experience of families. As Oude Maatman et al. pointed out, recognition of parents as primary caregivers by healthcare teams is crucial to the implementation of FCC. These investigators advocate efforts to first change the stance of healthcare professionals toward the family, where further changes should then gradually follow (1).

## Conclusion

Promoting FCC globally may prove a major challenge, particularly in vulnerable settings. Hospital managers play an important role in the FCC implementation process, which involves not only turning the principles of FCC into reality, but also taking steps to promote changes to current rules and routines, as well as devising policies supported by a culture that views the family as a unit to be cared for. Education is also critical in empowering families, typically not fully aware of their rights, and in strengthening their sense of autonomy. Bringing the education of healthcare teams up to date is an ongoing process, given the current deficiencies in family-related content in the curricula of undergraduate programs. It is crucial to provide healthcare teams and managers with knowledge about FCC that emphasizes the importance of interprofessional practices. The recommendation of Oude Maatman and colleagues to initiate FCC implementation with staff recognition of parents as primary caregivers constitutes a strategy for promoting FCC in NICUs in various healthcare systems.

## Author Contributions

All authors contributed to draft the first manuscript, read, and approved the final manuscript.

## Funding

Grant by CNPQ number 308281/2015-2.

## Conflict of Interest

The authors declare that the research was conducted in the absence of any commercial or financial relationships that could be construed as a potential conflict of interest.

## Publisher's Note

All claims expressed in this article are solely those of the authors and do not necessarily represent those of their affiliated organizations, or those of the publisher, the editors and the reviewers. Any product that may be evaluated in this article, or claim that may be made by its manufacturer, is not guaranteed or endorsed by the publisher.
